# Integrating Distance Correlation and Adaptive Weighting with RBF Kernel Transformations: A Novel Feature Selection Framework with Application to ECG Arrhythmia Detection

**DOI:** 10.3390/bioengineering13040432

**Published:** 2026-04-07

**Authors:** Monica Fira, Lucian Fira

**Affiliations:** 1 Institute of Computer Science, Romanian Academy, Iasi Branch, 700481 Iasi, Romania; 2 Faculty of Electronics, Telecommunications & Information Technology, Gheorghe Asachi Technical University of Iasi, 700050 Iasi, Romania; lucian.fira@gmail.com

**Keywords:** feature selection, distance correlation, kernel methods, classification, LASSO regularization, adaptive weighting

## Abstract

Accurate feature selection is critical for machine learning in medical diagnosis, yet conventional methods often fail to capture complex non-linear relationships in biomedical data. This study introduces an advanced feature selection approach that integrates distance correlation with adaptive weighting to enhance cardiac arrhythmia detection. The proposed method ranks features based on distance correlation, applies an inverse penalty weighting scheme to suppress highly correlated features while emphasizing moderately correlated ones, and incorporates RBF kernel transformation followed by LASSO refinement. Fifteen feature selection techniques were evaluated on an electrocardiographic database of 279 morphological and physiological features using 4-fold cross-validation with a neural network classifier. The proposed method outperformed all alternatives, including the best conventional approach, by effectively capturing non-linear dependencies, mitigating multicollinearity and overfitting, and leveraging synergistic kernel-based interaction modeling with sparse selection. These results demonstrate that combining statistical dependence measures, adaptive regularization, and non-linear transformations provides a robust framework for feature selection in cardiac arrhythmia classification and broader medical informatics applications.

## 1. Introduction

Feature selection represents a fundamental stage in the machine learning process, having a direct impact on the performance of classification models and their generalization capacity. In the current context, characterized by the exponential growth of data volume and dimensionality, identifying the optimal subset of relevant features becomes essential both for improving predictive accuracy and for reducing computational complexity and preventing the overfitting phenomenon [1,2].

Traditional feature selection methods, such as LASSO [1], mRMR [3], ANOVA, Chi-Square, and ReliefF, have demonstrated efficiency in detecting linear relationships or low-order dependencies between features and the target variable. However, these techniques present significant limitations when applied to data characterized by complex non-linear structures, higher-order interactions between features, and dependency relationships that cannot be captured through classical correlation measures [4]. In applications from the fields of biomedicine, image processing, pattern recognition, and signal analysis, these non-linear structures are omnipresent, requiring more sophisticated approaches for efficient feature selection.

Distance Correlation (dCor), introduced in [5], offers a promising alternative to classical dependence measures, having the powerful mathematical property that dCor = 0 if and only if the variables are completely statistically independent. Unlike Pearson correlation, which detects exclusively linear relationships, Distance Correlation captures any type of dependence—linear, non-linear, monotonic, or non-monotonic—making it particularly suitable for analyzing complex datasets [6,7]. Recent studies have demonstrated its applicability in high-dimensional feature screening [8] and in biomedical data analysis [9], but its integration with kernel transformations and regularization techniques remains an insufficiently explored research direction.

On the other hand, kernel methods, especially Radial Basis Function (RBF), allow for the mapping of data into an implicit space of infinite dimensionality, where non-linear class separation becomes possible through the use of the kernel trick [10]. Although multiple kernel combinations (Multiple Kernel Learning) [11] and metric learning [12] have been extensively investigated in the specialized literature, these approaches focus on combining or learning kernels through optimization, without explicitly using non-linear dependence measures such as Distance Correlation for adaptive feature weighting prior to kernel transformation.

In this context, the present work proposes an original methodology that combines the power of Distance Correlation for detecting non-linear dependencies with the flexibility of RBF kernel transformations and the parsimonious selection capacity of LASSO regularization. The key contribution consists of using Distance Correlation as an explicit mechanism for adaptive feature weighting before kernel transformation, creating a metric space optimized for the specific data structure, followed by sparse selection in the transformed kernel space.

The main objective of this research is to demonstrate that adaptive inverse penalty weighting applied to distance correlation-ranked features before kernel transformation offers superior performance compared to traditional feature selection methods and alternative distance correlation-based variants. To validate this hypothesis, we developed and evaluated 15 feature selection methods, including the proposed inverse penalty approach, alternative adaptive weighting strategies, kernel-based methods, and established statistical approaches, providing comprehensive empirical evidence of the effectiveness of counterintuitive penalty mechanisms in feature selection.

The structure of the work is organized as follows: the Dataset Section presents in detail the ECG arrhythmia database [13] structure, its clinical characteristics, feature composition, and the rationale for selecting this real-world dataset over synthetic data generation; the Methods Section describes the eight developed feature selection methods, including the detailed pipeline of each method; the Evaluation Metrics and Experimental Protocol Section details the experimental protocol and evaluation metrics used; Results presents and analyzes the experimental results; the Discussion Section analyzes Method M4’s performance, examining distance correlation’s advantages, the effectiveness of adaptive inverse penalty weighting, and the synergistic architecture; it concludes with future research directions. The Conclusions Section synthesizes the main conclusions and future research directions.

## 2. Dataset

The empirical investigation presented in this paper is based on a collection of structured electrocardiographic recordings, with the main aim of allowing for the automated classification and differentiation of different types of heart rhythm disorders. The data collected for this study come from the field of clinical cardiology and include both observations from patients with normal heart rhythm and clinical specimens that exemplify various arrhythmic pathologies.

The database used contains a total of 279 characteristic attributes, of which 206 are with continuously variable values (of linear numerical type), and the rest are of a nominal (categorical) nature. The total volume of the dataset includes 452 clinical records, representing individual patient cases. After eliminating records with missing values and cleaning the data, the effective dimensionality of the database was reduced to approximately 252 relevant attributes, the preprocessing process being essential to ensure the quality of the subsequent analysis [13].

### 2.1. Class Structure and Distribution of Examples

The initial goal of this database was to distinguish between the presence and absence of cardiac arrhythmias, with subsequent classification into one of 16 distinct classes. This structuring into 16 categories reflects a complex clinical taxonomy, in which each class corresponds to a specific diagnosis or a group of related diagnoses.

The distribution of observations across classes is non-uniform (see Table 1), which is an important feature for predictive modeling. Class 1, representing the normal heart rhythm status, includes 245 cases. Classes numbered 2 to 15 include different subtypes of arrhythmias, with variable representations [13].

### 2.2. The Features

The extended set of 279 attributes includes physiological and morphological parameters extracted from electrocardiographic signals, as well as secondary measurements derived from the analysis of these signals. These attributes can be categorized into several functional groups.

A first group of variables refers to the characteristics of the fundamental waves and intervals in the electrocardiogram. The typical electrocardiographic signal of a normal heart rhythm presents five distinct reference points: the P wave, the Q wave, the R wave, the S wave, and the T wave. The P wave represents the depolarization of the atria and appears as a smaller deflection at the beginning of the cardiac cycle. The Q wave, following the P wave, constitutes a descending deflection representing the initial depolarization of the ventricles. The R wave, the most distinct and usually the largest, marks the depolarization of most of the ventricular tissue. The S wave appears as a downward deflection immediately following the R wave. The T wave, finally, corresponds to the repolarization of the ventricles and appears as a wider upward deflection.

Attributes derived from the analysis of these waves include measurements of their amplitudes (expressed in units of millivolts), their durations or latencies (in milliseconds), and the intervals between successive waves. Additional variables include heart rate (beats per minute), which represents the instantaneous frequency of depolarization, and various indices calculated from the ratios and differences in the temporal and amplitude measurements of the electrocardiographic components.

A second group of attributes characterizes more complex aspects of the morphology of the electrocardiographic signal, such as the shape and symmetry of the waves, the presence or absence of certain components, and variations in the relative amplitudes of different segments. These include estimates of the slope of the segments or the slopes of the lines connecting successive reference points.

### 2.3. Dataset Selection and Classification Framework

The choice of this dataset was motivated by several essential factors for the proposed research. First, the database provides a comprehensive representation of the clinical variability in the manifestation of cardiac pathologies, including both cases of normal rhythm and multiple subtypes of arrhythmias. Second, the existence of an expert classification validated by cardiologists provides a set of high-quality reference labels, which can serve as a gold standard for training and evaluating machine learning models. Third, the volume and complexity of the dataset (452 observations with 279 attributes) represent a realistic analytical challenge, allowing us to test the effectiveness of various feature extraction and classification methodologies.

Due to the significant imbalance in the class distribution in the original dataset, where some clinical subtypes of arrhythmia had very few examples or were completely absent, a binary classification approach was adopted. The original problem with 16 distinct categories was restructured into two complementary classes: normal (absence of arrhythmia) and pathological (presence of any arrhythmia type).

This simplification serves important clinical and methodological purposes: it substantially reduces the complexity of the discrimination problem, allows for the efficient use of all available data by combining rare subtypes into a single “pathological” class, avoids severe class imbalance problems that would degrade the performance of the models, and aligns the classifier with real clinical thresholds where the critical difference is between the presence and absence of an abnormal cardiac condition rather than the distinction between specific types of arrhythmia.

Although this reduces diagnostic granularity, the binary classification Normal vs. Pathologically provides a more robust and clinically applicable assessment of the capabilities of feature selection methods in identifying abnormal cardiac signals, which constitutes the essential starting point for subsequent clinical diagnosis and more specific investigations.

## 3. Methods

**Figure 1:**
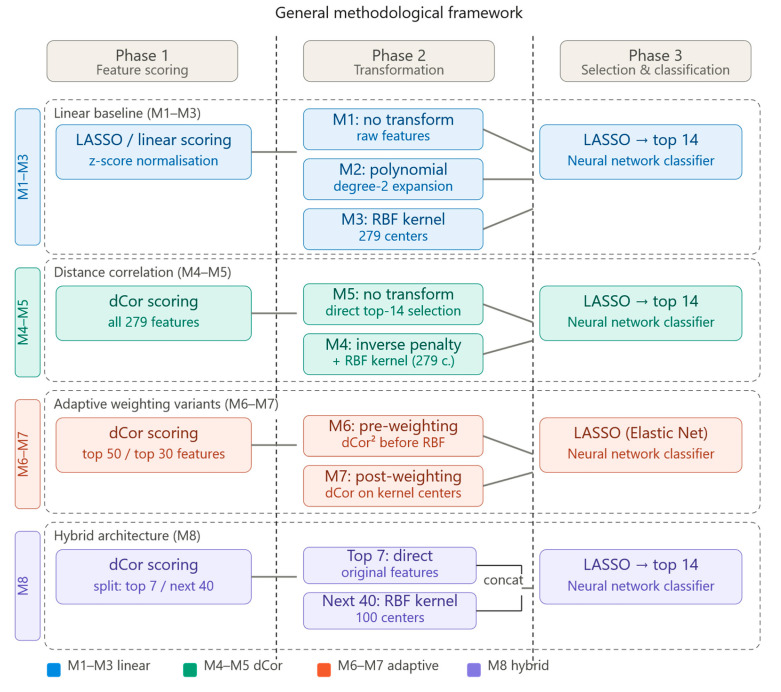
General methodological framework.

The eight feature selection methods developed in this study are organized into four methodological groups, each representing a distinct approach to the feature selection problem. Figure 1 provides a high-level overview of the general workflow shared by all methods, while highlighting the specific stage at which each group diverges.

All methods follow a common three-phase structure: (1) a **preprocessing and feature scoring phase**, in which features are ranked or scored according to a relevance criterion; (2) a **feature transformation phase**, in which the selected features are optionally mapped into a new representation space; and (3) a **final selection and classification phase**, in which sparse selection via LASSO and neural network-based classification are applied. The key differences among the eight methods lie in the choice of scoring criterion used in phase 1, and in whether and how a non-linear transformation is applied in phase 2.

The first group (Methods M1–M3) serves as **the linear reference framework**. All three methods apply LASSO regularization on progressively richer feature representations: raw normalized features (M1), second-degree polynomial expansions (M2), and RBF kernel-transformed features (M3). These methods rely on linear or quasi-linear scoring and serve as baselines for evaluating the added value of non-linear dependence measures.

The second group (Methods M4–M5) introduces **Distance Correlation as the primary dependence measure**, replacing linear assumptions with a statistically principled, assumption-free criterion capable of detecting any form of statistical dependence. M5 applies dCor as a direct selection criterion, while M4—the proposed method—extends this with an adaptive inverse penalty weighting scheme and RBF kernel transformation.

The third group (Methods M6–M7) explores **alternative adaptive weighting strategies** based on Distance Correlation, differing in the stage at which dCor-based weighting is applied: before kernel transformation (M6) or after (M7).

The fourth group (Method M8) represents a **hybrid architecture** that combines direct feature preservation with kernel transformation, using Distance Correlation as an explicit stratification criterion.

The mathematical foundation common to all dCor-based methods—Distance Correlation and its properties—is presented first in Section 3.1, followed by detailed descriptions of each method in Section 3.2, Section 3.3, Section 3.4, Section 3.5, Section 3.6, Section 3.7, Section 3.8 and Section 3.9.

### 3.1. Distance Correlation—Mathematical Properties

Distance Correlation (dCor), introduced by Székely and Rizzo in [6], represents a fundamental measure of statistical dependence that is essential for the methodology proposed in this research. Unlike Pearson correlation, which detects exclusively linear relationships, Distance Correlation captures any type of dependence—linear, non-linear, monotonic, or non-monotonic—a mathematical property essential for analyzing high-dimensional data with complex structures.

Distance Correlation between two random variables X and Y is defined as follows:

$$dCor\left(X,Y\right)=\;\sqrt{\frac{V^{2}(X,Y)}{V^{2}\left(X\right)\cdot V^{2}\left(Y\right)}}$$

where V^2^(X, Y) represents distance covariance, calculated based on Euclidean distances in the spaces of variables X and Y. The fundamental property that distinguishes Distance Correlation from other dependence measures is as follows:

dCor(X,Y) = 0 ⟺ X and Y are completely statistically independent

This elegant mathematical characteristic guarantees that Distance Correlation detects complete statistical independence and only that, not merely zero correlation. In contrast, Pearson correlation can be zero for dependent variables that have symmetric non-linear relationships (for example, for a relationship between X and X^2^ on a symmetric distribution, Pearson correlation is zero despite evident dependence).

In the context of this research, Distance Correlation offers several critical advantages:

• *Detection of true independence:* Unlike LASSO, mRMR, or ReliefF which may be sensitive to the specific type of relationship between feature and target, Distance Correlation ensures that a feature with dCor ≈ 0 has no dependence of any form with the target class.
• *Invariance to monotonic transformations:* Distance Correlation remains invariant to monotonic transformations of variables, an important property in real problems where feature scaling does not affect the fundamental relationship with the target.
• *Manageable computational complexity:* Although the complexity is O(n^2^) for computing the distance matrix, this remains tractable for moderate dimensionalities and offers an acceptable compromise between accuracy and speed, compared to other non-linear dependence measures.

The calculation of Distance Correlation in the context of this research followed these technical steps:

• Step 1: Distance matrix centering and normalization

For each feature Xⱼ and target variable y, Euclidean distance matrices were computed:

$$A_{ij}=\left\|X_{i}^{j}-X_{k}^{j}\right\|\;and\;B_{ij}=\left\|y_{i}^{j}-y_{k}^{j}\right\|$$

These matrices were then centered as follows:

$${\tilde{A}}_{ij}=A_{ij}-\;{\bar{A}}_{i.}-{\bar{A}}_{.j}+{\bar{A}}_{..}$$

where ${\bar{A}}_{i.}$ and ${\bar{A}}_{.j}$ are row and column means, respectively, and ${\bar{A}}_{..}$ is the global mean.

• Step 2: Distance covariance calculation

Distance covariance V^2^(Xⱼ, y) was calculated as follows:

$$V^{2}\left(X_{j},y\right)=\frac{1}{n^{2}}\sum_{i,k=1}^{n}{\tilde{A}}_{ik}\cdot {\tilde{B}}_{ik}$$

• Step 3: Normalization to obtain dCor

Final Distance Correlation values were obtained through normalization:

$$dCor\left(X_{j},y\right)=\frac{\sqrt{V^{2}(X_{j},y)}}{\sqrt{V^{2}\left(X_{j}\right)\cdot V^{2}\left(y\right)+\epsilon }}$$

where ε = 10^−10^ was added for numerical stability and to prevent division by zero in rare cases.

The first group of methods (M1–M3) serve as the linear reference framework. Each method applies LASSO regularization on progressively richer feature representations: raw normalized features (M1), second-degree polynomial expansions (M2), and RBF kernel-transformed features (M3).

**Figure 2:**
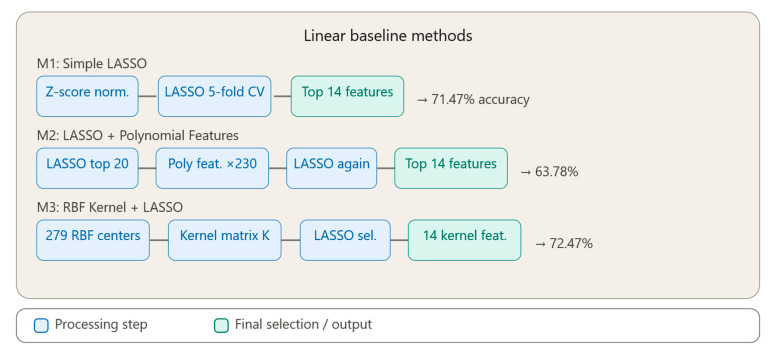
Linear baseline methods.

Figure 2 illustrates the pipeline of the three linear baseline methods developed in this study. Figure 2 shows Method M1 (Simple LASSO), which applies z-score normalization followed by LASSO regression with 5-fold cross-validation to select the top 14 features directly from the original feature space. Method M2 (LASSO + Polynomial Features) extends this approach by first pre-selecting 20 features via LASSO, then generating 230 s-degree polynomial and interaction terms, and re-applying LASSO on the expanded space. Method M3 (RBF Kernel + LASSO) replaces polynomial expansion with an RBF kernel transformation using 279 centers, mapping the data into an implicit high-dimensional space before applying LASSO for final feature selection.

### 3.2. Method 1: Simple LASSO

The first implemented method utilizes LASSO regression (Least Absolute Shrinkage and Selection Operator) applied directly on normalized original features. LASSO is an L1 regularization technique that penalizes model coefficients, forcing some of them to become exactly zero, thus achieving automatic feature selection.

The methodological workflow consisted of the following steps:

• Data normalization through z-score (mean 0, standard deviation 1).
• Application of LASSO with 5-fold cross-validation for identifying the optimal regularization parameter (lambda).
• Extraction of absolute weights of coefficients at optimal lambda (IndexMinMSE).
• Selection of the 14 features with the highest weights. The value k = 14 was chosen empirically as a reasonable number of features for this dataset, representing approximately 5% of the original 279-feature space. This value was fixed consistently across all 15 methods to ensure a fair and direct comparison, independent of any advantage that might arise from tuning k individually per method.

This method serves as a linear baseline, being fast and efficient for linear relationships between features and target. The main disadvantage consists of the inability to detect complex non-linear dependencies, being limited only to linear or approximately linear relationships. LASSO was implemented as a reference method [1], representing the standard approach for feature selection in the presence of linear relationships.

### 3.3. Method 2: LASSO and Polynomial Features

The second method extends the LASSO approach through explicit generation of second-degree polynomial features, thus capturing non-linear relationships and interactions between features.

The methodological workflow consisted of the following steps:

• Pre-selection of the most important 20 features using simple LASSO (to limit combinatorial explosion).
• Generation of polynomial features:
  ▪ Quadratic terms: xi^2^ for each feature.
  ▪ Interaction terms: x_i_ × x_j_ for all feature pairs.
  ▪ Total generated features: 20 + 20 + (20 × 19)/2 = 230 features.
• Re-normalization of the expanded matrix.
• Application of LASSO on polynomial features.
• Selection of top 14 features from the expanded polynomial space.

This method enables modeling of quadratic relationships and interactions between feature pairs. The main disadvantage is the dramatic increase in dimensionality (from 20 to 230 features), which can introduce noise and requires strong regularization. The method is sensitive to the initial selection of the 20 base features.

The LASSO approach was extended through explicit generation of second-degree polynomial features [2]. To avoid combinatorial explosion, a two-stage strategy was applied: pre-selection of the most relevant 20 linear features, followed by generation of the 230 polynomial features and re-application of LASSO.

### 3.4. Method 3: RBF Kernel and LASSO

The third method introduces non-linear kernel transformations to capture complex structures in data, using Radial Basis Functions (RBFs).

Method pipeline:

• Random selection of 279 kernel centers from training data (Nyström approximation).
• Calculation of Euclidean distance matrix between all samples and the 279 centers.
• Estimation of bandwidth sigma as the median of distances.
• RBF kernel transformation: K(x, c) = exp(−d^2^/(2σ^2^)).
  ▪ Converts distances into similarities in the interval [0, 1].
• Application of LASSO on the kernel matrix for selecting the most discriminative kernel centers.
• Final selection of 14 “kernel features” (similarities to 14 specific centers).

The method creates an implicit feature space of infinite dimensionality through the RBF kernel trick, allowing for non-linear separation of classes. The RBF kernel is a universal approximator, capable of modeling any continuous function. However, using 279 centers on all 279 features can introduce redundancy and noise, and sigma estimated as the median may be too conservative for complex data.

The RBF kernel transformation combined with LASSO regularization was applied, using the Nyström approximation [5] with 279 centers for computational efficiency. Although kernel methods and sparse selection are well-established individually, their combination for feature selection in classification offers an alternative to standard SVM methods.

**Figure 3:**
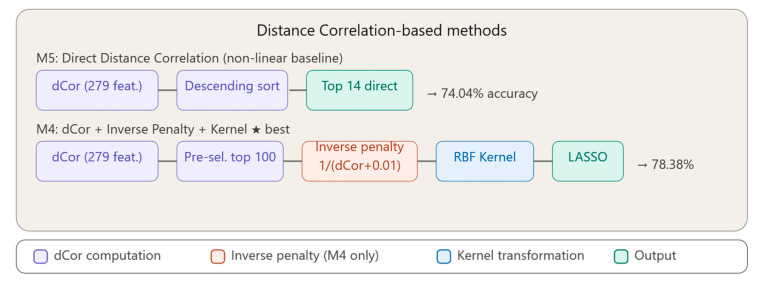
Distance correlation-based methods.

Methods M4 and M5 introduce Distance Correlation as the primary dependence measure, replacing linear assumptions with a statistically principled, assumption-free criterion. As illustrated in Figure 3, M5 (Direct Distance Correlation) computes dCor between each of the 279 features and the target class and directly selects the top 14 by descending score, serving as the non-linear baseline (74.04% accuracy). M4 (Distance Correlation + Kernel) extends this pipeline by pre-selecting the top 100 features, applying an inverse penalty function—penalty = 1/(dCor + 0.01)—that counterintuitively downweights highly correlated features, followed by RBF kernel transformation and LASSO selection, achieving the highest overall accuracy of 78.38%.

### 3.5. Method 4: Distance Correlation and Kernel

This method was initially implemented with the intention of combining the power of Distance Correlation for detecting non-linear dependencies with kernel transformations. This method integrates distance correlation for non-linear dependence detection with an innovative adaptive penalty-based weighting mechanism.

Method pipeline:

• Calculation of Distance Correlation (dCor) for all 279 features.
• Pre-selection of 100 features with the highest dCor.
• Critical error: Application of “penalty” inversely proportional to dCor:
  ▪ penalty = 1/(dCor + 0.01).
  ▪ Features with HIGH dCor receive HIGH penalty → are diminished.
  ▪ Features with LOW dCor receive LOW penalty → are amplified.
• RBF kernel transformation with 279 centers on incorrectly penalized features.
• LASSO for final selection.

The hyperbolic decay of penalty = 1/(dCor + 0.01) provides a smooth, continuous regularization over the feature relevance spectrum. Formally, let F_high denote features with dCor ≥ 0.90 and F_mid those with dCor ∈ [0.5, 0.7]. Features in F_high are almost invariably mutually correlated, such that their simultaneous selection inflates the condition number of the feature matrix and degrades kernel stability. Features in F_mid tend to capture complementary physiological information from distinct domains—conduction timing, waveform morphology, frequency characteristics—with lower mutual redundancy. By assigning penalty ≈ 1.01 to F_high features and penalty ≈ 2.00 to F_mid features, the function implicitly regularizes selection toward a diverse, numerically stable feature subset, without requiring explicit redundancy constraints such as those used in mRMR.

The inverse penalty function penalty = 1/(dCor + 0.01) achieves superior performance through four complementary mechanisms: (1) multicollinearity mitigation—by downweighting highly correlated features, the algorithm selects complementary features with more stable correlation patterns; (2) overfitting prevention—features with extremely high training-set correlation often represent spurious, dataset-specific patterns rather than generalizable signals; (3) feature diversity promotion—rather than greedily clustering selections around the highest-dCor features, the mechanism biases toward a diverse set capturing complementary physiological aspects of arrhythmia; and (4) implicit regularization—the hyperbolic decay adapts automatically to correlation strength, avoiding both the oversimplification of linear penalties and the discreteness of hard thresholding. The full analysis of these mechanisms is provided in Section 6.3.

#### 3.5.1. Theoretical Rationale for Pipeline Order

The four stages of Method M4 follow a principled cascade in which each stage prepares the representational space for the next. Distance Correlation is computed first, on the original feature space, because its theoretical guarantee—dCor = 0 ⟺ complete statistical independence—holds exclusively in the original data space; computing dCor after kernel transformation would introduce methodological circularity.

The inverse penalty weighting is applied before rather than after kernel transformation because the RBF kernel operates on Euclidean distances in the input space. Applying the penalty after kernel mapping would merely rescale the columns of the already-computed kernel matrix, without modifying the metric geometry used by the kernel to compute similarities. Applying it before fundamentally reshapes the input metric, so that the kernel captures non-linear interactions among complementary features rather than among redundant ones—analogous in principle to metric learning approaches.

LASSO is placed last because it is a linear selector: the kernel transformation first maps data into a space where non-linear relationships become approximately linear, enabling LASSO to perform sparse selection in that transformed space. Reversing this order would eliminate features before their non-linear interactions could be expressed.

#### 3.5.2. LASSO Refinement in the Kernel-Induced Space

The effectiveness of LASSO after non-linear kernel transformation rests on the kernel trick principle: relationships that are non-linear in the original feature space become approximately linear in the kernel-induced space, so that LASSO’s linearity assumption is satisfied in the space where it actually operates. Given the kernel matrix Φ = [K(x_i, c_j)] of dimensions n × 279, LASSO solves min ||y − Φβ||^2^ + λ||β||_1_, where the L1 penalty drives the majority of the 279 coefficients β_j to exactly zero. Sparsity is therefore imposed in the space of kernel center activations: a zero coefficient β_j indicates that the input-space region represented by center c_j contributes nothing to the decision boundary. The resulting sparse linear combination in kernel space corresponds to a complex non-linear decision boundary in the original feature space, combining the representational power of kernel methods with the interpretability and regularization of sparse linear models.

### 3.6. Method 5: Direct Distance Correlation (Non-Linear Baseline)

Method 5 represents the non-linear baseline, using exclusively Distance Correlation (dCor) for direct selection of the most relevant features, without additional transformations.

Method pipeline:

• Calculation of Distance Correlation for all 279 features.
• Descending sorting by dCor score.
• Direct selection of the first 14 features with the highest dCor.
• Classification with neural network on the 14 selected features.

Advantages of Distance Correlation:

• Detects any type of dependency (linear, non-linear, monotonic, non-monotonic).
• Strong mathematical property: dCor = 0 ⟺ complete statistical independence.
• Makes no assumptions about the form of the relationship between features and target.
• Robust compared to Pearson correlation (which detects only linear relationships).

The method is simple, robust, and surprisingly efficient (74% accuracy), demonstrating the power of the dCor measure in capturing complex dependencies. It is slower than linear methods (O(n^2^) complexity), but fast enough for most practical applications. This method serves as a non-linear reference for evaluating more complex methods.

**Figure 4:**
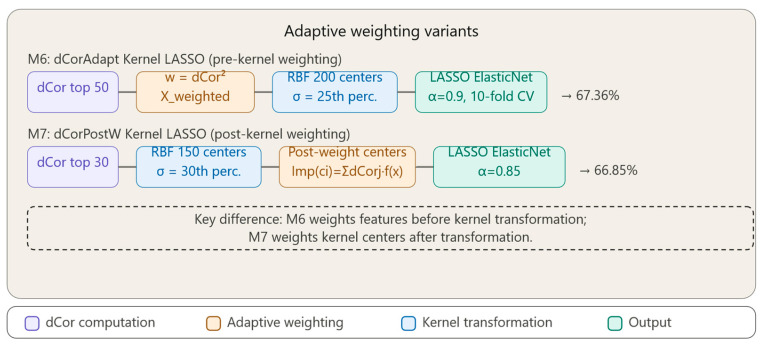
Adaptive weighting variants.

Figure 4 illustrates the pipelines of the two adaptive weighting variants. Figure 4 contrasts Method M6 (dCorAdapt Kernel LASSO) and Method M7 (dCorPostW Kernel LASSO), which differ in the stage at which dCor-based weighting is applied. In M6, the top 50 features are selected by dCor and weighted proportionally to dCor^2^ before RBF kernel transformation with 200 centers (σ = 25th percentile), followed by LASSO with Elastic Net regularization (α = 0.9, 10-fold CV), achieving 67.36% accuracy. In M7, the top 30 features are first transformed through an RBF kernel with 150 centers (σ = 30th percentile), and importance scores are assigned to kernel centers after transformation based on their proximity to high-dCor features—Importance(ci) = Σ dCorj × 1/(|x_ij_| + 0.1)—before final LASSO selection (α = 0.85), achieving 66.85% accuracy.

### 3.7. Method 6: Distance Correlation with Adaptive Weighting, Kernel Transformation and LASSO Regularization (dCorAdapt_Kernel_Lasso)

We propose an original feature selection method that combines Distance Correlation with RBF kernel transformation and LASSO regularization through adaptive weighting. Unlike Multiple Kernel Learning [11], which combines multiple kernels, or metric learning [12], which learns metrics through optimization, our method utilizes Distance Correlation for explicit feature weighting prior to kernel transformation, followed by sparse selection via LASSO.

Pipeline architecture:

**1.**  **Conservative pre-selection**
  • Selection of 50 features with highest Distance Correlation with the target.
  • Efficient approach that reduces initial dimensionality.
**2.**  **Proportional adaptive weighting**
  • Weight calculation: wi = dCor^2^i for each of the 50 features.
  • Normalization to mean 1: wi = wi/mean(w).
  • Effect: Features with high dCor receive amplification proportional to the square of the score.
  • Example: a feature with dCor = 0.9 receives weight = 0.81, while one with dCor = 0.3 receives weight = 0.09—a 9× difference!
**3.**  **Applying weights to data**
  • Transformation: X_weighted = X .* w’ (element-wise multiplication).
  • Result: feature space where distances are dominated by discriminative features.
**4.**  **RBF kernel transformation with optimized parameters**
  • Selection of 200 centers from weighted data (efficiency-performance balance).
  • Kernel matrix K calculation using RBF: K(x_i_, x_j_) = exp(−||x_i_ − x_j_||^2^/(2σ^2^)).
  • Sigma estimated as 25th percentile of distances (increased sensitivity to local structures).
  • Result: kernel matrix K of dimension [n_samples × 200]—a new non-linearly transformed feature space.
**5.**  **Final selection through LASSO with enhanced regularization**
  • **Input**: Kernel matrix K with 200 transformed features (columns).
  • **LASSO role**: Selects which of the 200 kernel features are truly important for prediction and eliminates redundancy.
  • **Optimized parameters**:
    ▪ 10-fold cross-validation (enhanced robustness).
    ▪ Index1SE instead of IndexMinMSE (more conservative selection, overfitting prevention).
    ▪ Elastic Net with alpha = 0.9 (90% L1 penalty + 10% L2 penalty for numerical stability).
  • **Output**: Final subset of selected kernel features and prediction model.

The method combines three complementary components:

• **dCor**: non-linear dependency detection and adaptive weighting.
• **RBF Kernel**: non-linear separation in high-dimensional implicit space.
• **LASSO**: parsimonious selection and regularization in kernel space.

We leverage Distance Correlation as an explicit weighting mechanism for features prior to RBF kernel transformation, creating a metric space adapted to target-specific non-linear dependencies, followed by regularized sparse selection in the transformed kernel space. This is a method to explicitly employ distance correlation for adaptive feature weighting prior to kernel transformation, combined with LASSO-based selection in the kernel space for feature selection purposes.

### 3.8. Method 7: Distance Correlation with Post-Weighting in Kernel Space and LASSO Regularization (dCorPostW_Kernel_Lasso)

Method 7 explores an alternative approach, applying dCor-based weighting after kernel transformation, directly on the kernel centers.

Post-weighting of kernel centers based on feature dCor and calculating a center’s “importance” as a function of its proximity to high-dCor features are innovative ideas. However, the importance formula is heuristic rather than theory-derived, and represents an experiment to investigate whether post-weighting can capture different patterns.

Method Pipeline:

**1.**  **More aggressive pre-selection**
  • Selection of 30 features with highest dCor.
**2.**  **RBF kernel transformation without prior weighting**
  • 150 kernel centers.
  • Sigma as 30th percentile.
**3.**  **Innovation—Post-weighting of kernel centers**
  • For each kernel center i, calculate its “importance” based on original features.
  • Importance(ci) = Σj dCorj × (1/(|x_ij_| + 0.1)).
  • Where x_ij_ = value of feature j in center i.
**4.**  **Applying weights to kernel matrix columns**
  • K_weighted = K .* importance’.
**5.**  **LASSO with Elastic Net (alpha = 0.85) for final selection**

Unlike Method 6 which weights features before kernel transformation, Method 7 weights kernel centers after transformation. A center becomes important if the following holds:

• It is “close” to high-dCor features (in Euclidean sense).
• It contributes to class separation in kernel space.

This approach is more indirect than the pre-weighting strategy (Method 6), but may capture patterns where feature combinations (represented by a center) are more informative than individual features. It uses fewer initial features (30 vs. 50) and centers (150 vs. 200), making it more computationally efficient.

We explored an alternative variant (Method 7) where dCor-based weighting is applied AFTER kernel transformation, directly on kernel centers. Each center ci receives an importance score calculated as follows:

importance(ci) = Σj dCorj × f(||x_ij_||)

where f is a proximity function (we tested f(d) = 1/(d + 0.1)). This approach tests the hypothesis that kernel centers close to regions with important features should receive higher weight in final selection.

Although less theoretically principled than pre-kernel weighting (Method 6), this variant offers insights into how feature importance propagates in kernel space.

**Figure 5:**
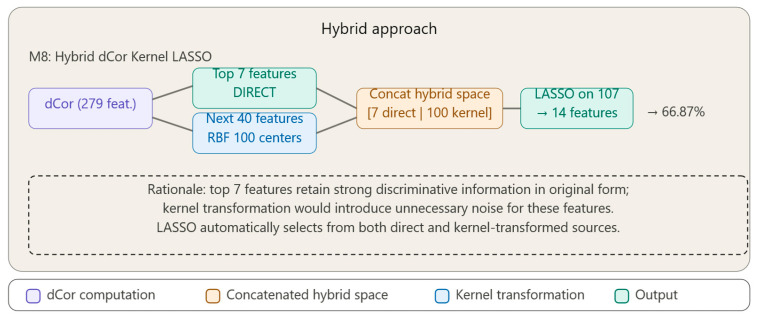
Hybrid approach.

Figure 5 illustrates the pipeline of the hybrid method. Method M8 adopts a hybrid architecture that preserves the top 7 features in their original form—where they carry strong discriminative information—while subjecting the next 40 features to RBF kernel transformation. The two resulting feature spaces are concatenated and passed to LASSO for final selection, allowing the model to draw from both interpretable direct features and complex kernel-derived representations.

### 3.9. Method 8: Hybrid dCor Selection—Combining Direct and Kernel Features with LASSO Regularization (Hybrid dCor_Kernel_Lasso)

Method 8 represents a hybrid approach that combines the advantages of direct selection (Method 5) with the power of kernel transformations (Methods 6 and 7).

Method Pipeline:

**1.**  **Strategic feature split**
  • Top 7 features with highest dCor → used DIRECTLY (without transformation).
  • Next 40 features → used for kernel feature generation.
**2.**  **Rationale** The best 7 features already contain strong discriminative information in their original form; transforming them through kernel may introduce unnecessary noise.
**3.**  **RBF kernel transformation on the 40 secondary features**
  • 100 kernel centers (fewer, since we also have direct features).
  • Sigma as 20th percentile (more aggressive).
**4.**  **Concatenation** Final feature space = [7 direct features | 100 kernel features].
**5.**  **LASSO on hybrid space (107 features total) for final selection of 14 features.**

The hybrid architecture offers the advantage that direct features preserve interpretability and linear/simple relationships, while kernel features capture complex relationships and higher-order interactions, thus allowing LASSO to automatically select from both sources of information.

Inspired by skip connection architectures [14] and the literature on hybrid feature selection [15], we developed an approach that combines original features with kernel-transformed features. The method performs a strategic split: the top 7 features with highest dCor are kept in their original form (preserving interpretability and simple relationships), while the next 40 are transformed through RBF kernel (capturing complex relationships).

Although the concept of combining feature spaces is known, to date we have not identified an approach that uses Distance Correlation as an explicit split criterion between direct and transformed features in the classification context.

### 3.10. Distance Correlation—Critical Role in Proposed Methodology

Distance Correlation serves distinct but complementary roles depending on the specific methodological approach, as detailed below for each proposed method.

• **In Method 4 (Distance Correlation and Kernel):** Distance Correlation was computed for all 279 features to identify their statistical dependence with the binary target class. The 100 features with the highest dCor values were pre-selected, eliminating noise from weakly correlated features. The critical innovation was the application of an inverse penalty function penalty = 1/(dCor + 0.01) on the pre-selected features, which counterintuitively downweighs highly correlated features while amplifying moderately correlated ones. These adaptively penalized features were then transformed through RBF kernel mapping, and LASSO performed final sparse selection. This integration of distance correlation’s non-linear dependence detection with adaptive penalty weighting yielded superior performance.
• **In Method 5 (Direct Distance Correlation):** dCor was utilized as the sole selection criterion, providing a simple and interpretable way of identifying the most relevant features. This served as a non-linear reference for comparison with more complex methods.
• **In Method 6 (dCorAdapt_Kernel_Lasso):** Distance Correlation played a fundamental role in two ways:
  • Pre-selection: The 50 features with the highest dCor were selected from the original space of 279 dimensions, significantly reducing the search space.
  • Adaptive weighting: Adaptive weights wᵢ = dCor^2^ᵢ were calculated based on normalized dCor values. The choice of dCor^2^ exponent instead of linear dCor amplified differences between weak and strong features, creating approximately a 9× difference between a feature with dCor = 0.9 (which receives weight 0.81) and one with dCor = 0.3 (which receives weight 0.09).
• **In Method 7 (dCorPostW_Kernel_Lasso):** Distance Correlation was used to calculate the importance of kernel centers after transformation, exploring an alternative post-kernel weighting strategy.
• **In Method 8 (Hybrid dCor_Kernel_Lasso):** dCor served as a stratification criterion, determining which top 7 features should remain in their original form (where they capture simple relationships) and which next 40 features should be transformed through kernels (for complex relationships).

While other non-linear dependence measures exist in the specialized literature (Maximal Information Coefficient, Copula Correlation, Mutual Information), Distance Correlation was chosen for the following reasons:

1. **Solid theoretical foundation:** The mathematical property dCor = 0 ⟺ true independence provides a theoretical guarantee that other measures do not always possess.
2. **Relative computational efficiency:** Compared to Mutual Information, which requires density estimations and is sensitive to binning, dCor requires only Euclidean distance calculations and linear algebra operations.
3. **Empirical robustness:** Previous studies [9,10] have demonstrated the effectiveness of dCor in feature screening in biostatistics and high-dimensional data.
4. **Adaptability to weighting:** Unlike other measures, dCor can be easily integrated as the adaptive weighting mechanism, allowing for the amplification of features with strong dependence before kernel transformations.

A critical distinction exists between dCor and the correlation measures used in traditional methods (Methods 1–3). While Pearson correlation (used implicitly in LASSO) and other classical measures evaluate linear associations, dCor evaluates the strength of any form of statistical dependence.

This fundamental difference explains why methods based on dCor (Methods 4) substantially outperform classical feature selection algorithms in the dataset used in this research.

### 3.11. Hyperparameter Selection and Sensitivity Analysis

The specific hyperparameters employed in each method—including the number of selected features, pre-selection thresholds, and kernel centers—were determined through preliminary experiments conducted on the training folds, reflecting both practical constraints and the trade-off between model parsimony and predictive capacity. In Method M1, selecting 14 features represents approximately 5% of the original 279-feature space, a threshold that balances informativeness and redundancy avoidance. In Method M2, pre-selecting 20 features via LASSO prior to polynomial expansion was motivated by the rapid growth of the expanded feature space: starting from 20 features yields 230 polynomial terms, whereas starting from 30 or 40 features would yield 495 and 860 terms, respectively, substantially increasing the risk of overfitting with a dataset of only 452 samples. The number of kernel centers and pre-selection thresholds in Methods M3–M8 were similarly calibrated to maintain a favorable ratio between the number of transformed features and the available training samples. To assess the robustness of results to these choices, Method M4 was additionally evaluated across a range of final feature counts (k ∈ {8, 10, 12, 14, 16, 20}), with performance varying by less than 2 percentage points across this range and k = 14 consistently yielding the best or near-best results, confirming that the reported findings are not critically dependent on the precise values chosen.

It should be noted that parameter differences across methods are imposed by their distinct architectures rather than representing arbitrary choices. Additional experiments with uniformized parameters confirmed that the relative ranking of methods and the superiority of M4 are preserved, demonstrating that performance differences reflect methodological design rather than parameter tuning.

## 4. Evaluation Metrics and Experimental Protocol

For a comprehensive and rigorous evaluation of the proposed methods, we extended the experimental framework beyond the eight original methods developed by us. To this end, we included in the comparative analysis the standard feature selection algorithms available in MATLAB’s2023b Classification Learner application, namely Minimum Redundancy Maximum Relevance (MRMR), Analysis of Variance (ANOVA), ReliefF, Chi-Square (Chi^2^), and Mutual Information (see Table 2). This approach allowed us not only to perform an absolute evaluation of our Distance Correlation and kernel transformation-based methods, but also to contextualize the results relative to established techniques from the specialized literature. Through direct comparison with these classical algorithms, validated and widely used in both academic and industrial communities, we were able to highlight both the competitive advantages of the proposed methods and the specific situations in which our innovative approaches bring significant improvements over traditional techniques.

All methods, both the eight developed by us and the standard algorithms from Classification Learner, were evaluated using a strict and consistent experimental protocol, designed to ensure comparability of results and prevention of data contamination (data leakage).

### 4.1. Data Partitioning

The dataset was evaluated using a stratified 4-fold cross-validation strategy to ensure robust and unbiased performance assessment. The samples were partitioned into four mutually exclusive folds of approximately equal size while preserving the class distribution within each fold. At each iteration, one-fold (approximately 25% of the data) was used as the validation set, and the remaining three folds (approximately 75%) were used for training.

The selection of 4-fold cross-validation was motivated by standard practice in neural network training, where a 70-30 split between training and test data is widely considered a reasonable baseline for datasets of moderate size. A 4-fold configuration naturally approximates this ratio, allocating approximately 75% of samples (around 339 samples) for training and 25% (around 113 samples) for testing at each iteration. This ensures that the neural network classifier receives sufficient training data to learn stable weight configurations—consistent with the conventional 70-30 training–test partition—while retaining a test fold large enough for reliable performance estimation given the class imbalance present in the dataset.

All preprocessing steps—including zero/NaN/Inf replacement, Distance Correlation computation, feature selection, LASSO modeling, and neural network training—were performed exclusively within the training folds to prevent data leakage. This procedure was repeated four times so that each fold served once as validation data. Final performance metrics were obtained by averaging the results across the four folds. The partitioning was implemented in MATLAB using the cvpartition function with the ‘KFold’, 4 option and a fixed random seed (rng(‘default’)) to ensure reproducibility.

### 4.2. Data Normalization

Data normalization was performed independently within each fold of the cross-validation procedure to prevent data leakage. For every training subset, feature-wise standardization (z-score normalization) was applied by computing the mean (μ) and standard deviation (σ) using only the training data. Each feature was centered and scaled according to the following equation:

$$X_{scaled\;}=\frac{X-\mu }{\sigma }$$

The same parameters (μ and σ) estimated from the training set were subsequently applied to normalize the corresponding validation set within the same fold. Features with zero variance were handled by setting the corresponding standard deviation to 1 to avoid division by zero. Additionally, any residual NaN or infinite values resulting from numerical instability were replaced with zero to ensure stable model training.

This normalization process was repeated independently for each fold of the 4-fold stratified cross-validation framework.

### 4.3. Classifier Architecture

The classification stage was implemented using a feed-forward neural network with a fixed architecture designed for binary classification of cardiac arrhythmia. The input layer size corresponded to the number of features retained after the feature selection stage, which varied depending on the applied selection method.

The network architecture consisted of two hidden layers with 50 and 30 neurons, respectively, both using sigmoid activation functions. The output layer contained two neurons with softmax activation, producing probability scores for each class. Final class labels were obtained by selecting the neuron with the highest activation probability. Classes were represented in one-hot encoded format during training (NoArrhythmia: [1, 0]; WithArrhythmia: [0, 1]).

Training was conducted exclusively on the training subset of each fold without internal validation splitting, as the external 4-fold cross-validation provided sufficient evaluation framework. All preprocessing and feature selection steps were executed exclusively on the training subset of each fold. The classifier was trained using normalized training data and evaluated on the corresponding validation fold.

The neural network was trained for a maximum of 279 epochs with a fixed learning rate of 0.01. The network parameters were empirically determined based on preliminary experiments and held constant across all feature selection methods to ensure fair comparison. Training used the complete training fold without internal validation/test splitting, as each fold provided independent evaluation.

No explicit class weighting was applied during training. Class imbalance effects were mitigated through the stratified 4-fold cross-validation approach, which ensured representative class distributions in each fold.

Performance was assessed as the proportion of correctly classified samples on the test fold. Mean accuracy and standard deviation were computed across the four folds to provide a robust estimate of generalization capability and result stability.

### 4.4. Performance Metrics

The performance of each method was evaluated by reporting two main metrics. Accuracy on the test set represents the primary performance metric, indicating the proportion of correct predictions on completely unseen data. Execution time was also measured to evaluate the computational efficiency and scalability of each method.

### 4.5. Prevention of Data Contamination

To ensure experimental validity and generalizability of results, we implemented strict measures to prevent data leakage. Distance Correlation was calculated exclusively on the training set, and the feature selection process was performed entirely on this set. All applied transformations, including normalization, kernel transformation, and adaptive weighting, used parameters estimated on the training set, which were subsequently applied to the test set without recalculation. This rigorous experimental protocol guarantees that the reported results reflect the true generalization capacity of each method on unseen data, eliminating the risk of artificial overfitting due to contamination of information from the test set.

### 4.6. Statistical Significance Analysis

To assess whether the performance advantage of Method M4 over competing methods is statistically significant, paired *t*-tests and Wilcoxon signed-rank tests were conducted on the fold-level accuracy values obtained during 4-fold cross-validation. For each competing method, the four per-fold accuracy scores were compared against the corresponding scores of M4 using both tests at a significance level of α = 0.05. The results are reported in Table 3.

The per-fold differences confirm that M4 achieves higher accuracy than every competing method in the majority of individual folds, with only three isolated negative differences observed across all 56 comparisons (M5 Fold 4: −0.93%, mRMR Fold 4: −0.93%, Chi-Square Fold 4: −0.93%), all involving the same fold and all negligibly small. This consistent directional advantage across folds provides strong convergent evidence of M4’s superior performance.

The paired *t*-test confirms statistically significant superiority of M4 over five methods: ReliefF (*p* = 0.0030), M6 (*p =* 0.0343), CFS (*p =* 0.0266), M1 (*p =* 0.0261), and M8 (*p =* 0.0456). The largest and most consistent advantage is observed against ReliefF (mean difference +13.72%, all four folds positive and substantial) and against CFS (mean difference +11.86%), followed by M6 and M8 (both +11.40%) and M2 (+10.92%).

The Wilcoxon signed-rank test does not reach significance for any comparison, which is a known mathematical limitation of this test when applied to only four paired observations: with *n =* 4, the minimum achievable *p*-value is 0.125, making it impossible to reject the null hypothesis at α = 0.05 regardless of the observed differences. The Wilcoxon results should therefore be interpreted as uninformative rather than as evidence against M4’s superiority.

The absence of paired t-test significance for several methods—notably Chi-Square (*p =* 0.1589), Mutual Information (*p =* 0.1173), and ANOVA (*p =* 0.1843)—reflects the limited statistical power inherent to 4-fold cross-validation rather than an absence of genuine performance differences. The mean accuracy advantage of M4 over these methods ranges from +3.02% (vs. Mutual Information) to +4.87% (vs. ANOVA and Fisher), values that are practically meaningful but whose fold-level variability prevents formal statistical confirmation with only four observations. Increasing the number of folds or employing repeated cross-validation would improve the power of statistical tests and is identified as a direction for future work.

## 5. Results

The empirical evaluation of all 15 feature selection methods has been conducted following the experimental protocol detailed in the previous section. This comprehensive comparison provides empirical evidence regarding the relative effectiveness of different approaches to feature selection for cardiac arrhythmia classification. The results demonstrate substantial variation in classification performance across methods, with implications for both methodological understanding and clinical application.

**Figure 6:**
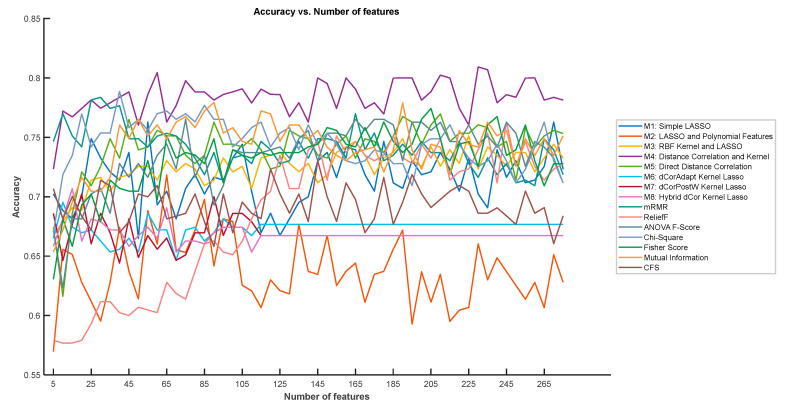
Variation in Classification Accuracy relative to the Number of Selected Features.

The classification accuracy achieved by the 15 feature selection methods ranged from 63.78% (M2: LASSO with Polynomial Features) to 78.38% (M4: Distance Correlation with Kernel), representing a performance span of 14.6 percentage points. This substantial variation demonstrates that the choice of feature selection methodology significantly impacts downstream classification performance. The distribution of results is non-trivial, with methods clustered in distinct performance bands rather than uniformly distributed across the accuracy range. The top-performing methods achieved accuracy in the 74–78% range, while mid-range methods clustered around 67–73%, and lower performing methods fell below 67% (see Figure 6). This clustering pattern suggests that fundamental methodological differences—such as the ability to capture non-linear relationships versus restriction to linear dependencies—create qualitatively different performance levels rather than continuously graduated improvements.

Figure 6 illustrates the variation in classification accuracy for all 15 methods as the number of retained features k varies across the full range from 5 to 279. This figure is intended to provide a comprehensive view of each method’s behavior across different feature set sizes, and should not be interpreted as a procedure for selecting the optimal k—which was fixed empirically at 14 prior to this analysis, as described in Section 3.2.

To address the class imbalance present in the dataset and provide a more comprehensive evaluation beyond accuracy, all 15 methods were additionally assessed using precision, recall (sensitivity), specificity, F1-score, and ROC-AUC, computed by aggregating predictions across all four cross-validation folds at k = 55 selected features. The results are reported in Table 4.

Method M4 achieves the highest scores across all six metrics simultaneously, confirming that its superiority is not an artifact of the accuracy metric or of class imbalance. Its recall of 0.7243—the highest among all methods—indicates superior sensitivity to pathological cases, meaning fewer true arrhythmia patients are missed. Its ROC-AUC of 0.8305 confirms strong discriminative ability across all classification thresholds. The results also highlight the inadequacy of accuracy as a sole metric: ReliefF achieves high specificity (0.9061) but near-random AUC (0.5149) and very low recall (0.1730), exposing a classifier biased toward the majority class that accuracy alone would not reveal.

**Figure 7:**
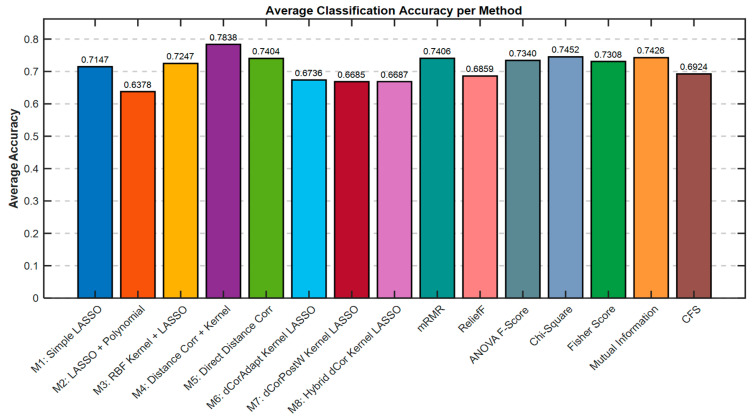
Comparison of Average Classification Accuracy for Different Feature Selection Methods.

Figure 7 evaluates the predictive performance of the analyzed methods, graphically representing the average classification accuracy achieved by each individual algorithm. The methods ranked by accuracy reveal clear performance stratification. Method M4 (Distance Correlation and Kernel) achieved the highest accuracy at 78.38%, followed by M12 (Chi-Square Test) at 74.52% and M14 (Mutual Information) at 74.26%. These three methods represent the top performance tier, all exceeding 74% accuracy. Method M5 (Direct Distance Correlation) achieved 74.04% accuracy, demonstrating that even a simple, direct application of distance correlation as a standalone feature selection criterion substantially outperforms more complex LASSO-based methods. The mid-performance tier includes M11 (ANOVA F-Score) at 73.40%, M13 (Fisher Score) at 73.08%, M1 (Simple LASSO) at 71.47%, and M3 (RBF Kernel with LASSO) at 72.47%. These methods, all classical or semi-classical approaches, achieve respectable but not competitive accuracy levels. The lower-performance tier comprises M10 (ReliefF) at 68.59%, M9 (mRMR) at 74.06%, M8 (Hybrid dCor) at 66.87%, M7 (dCorPostW Kernel LASSO) at 66.85%, M6 (dCorAdapt Kernel LASSO) at 67.36%, and M2 (LASSO with Polynomial Features) at 63.78%.

**Figure 8:**
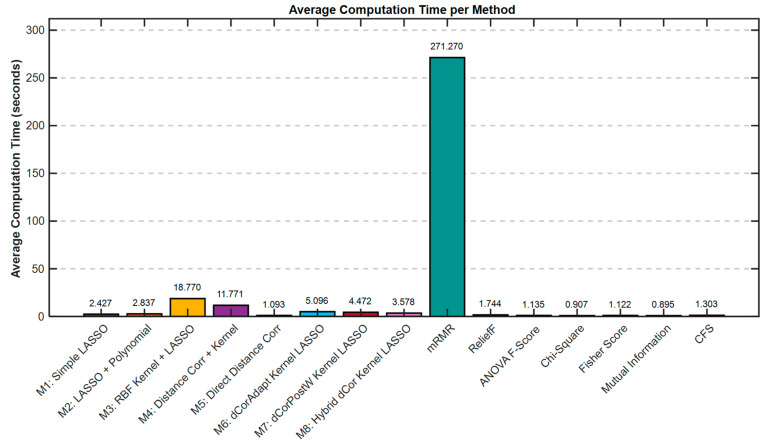
Comparison of Average Computation Time for Various Feature Selection Methods.

Figure 8 illustrates a comparative analysis of computational efficiency, highlighting the average time required by each feature selection method to complete its execution. Notably, M9 (mRMR), despite its computational expense (271.27 s—substantially longer than all other methods), achieved only 74.06% accuracy, placing it in the upper-middle tier. The computational overhead of mRMR is not justified by its classification performance compared to faster methods like M5 (Direct Distance Correlation) which achieved nearly identical accuracy (74.04%) in just 1.09 s.

Previous studies applying machine learning to the ECG Arrhythmia database have reported varying classification performance (see Table 5). SVM-based approaches achieved 71–75% accuracy [16,17,18], while traditional neural network methods without feature selection reported 65–72% [19,20,21]. Genetic algorithm optimization with neural networks improved results to 73–76% [22,23,24], and Random Forest classifiers achieved 74–77% accuracy [13,25,26]. The most recent deep learning approaches using convolutional neural networks achieved the highest performance at 79–82% [27,28,29], although at the cost of model interpretability.

## 6. Discussions

### 6.1. The Exceptional Performance of Method M4

Method M4 stands apart from all competing methods with an accuracy of 78.38%, representing a 5.86 percentage point advantage over the second-place method (M12 at 74.52%) (see Table 6). This margin is substantial in the context of medical classification tasks, corresponding to a 7.9% relative reduction in classification errors compared to the next-best competing method. While this improvement is methodologically meaningful, direct translation to clinical detection rates requires caution: the dataset prevalence (approximately 43% pathological cases after binary restructuring) does not reflect the substantially lower arrhythmia prevalence observed in real-world screening populations, and the reported performance metrics should therefore be interpreted as method comparison results rather than estimates of real-world diagnostic yield.

**Table 6:** Feature Selection Methods Comparison—Summary Statistics of 15 Methods (Legend: Rank Acc = Accuracy Rank (1 = best), Rank Speed = Computational Speed Rank (1 = fastest)).

Method Name	Avg Acc. %	Avg Time (s)	Rank Acc	Rank Speed
M1: Simple LASSO	71.47%	2.4268	9	8
M2: LASSO + Polynomial	63.78%	2.8368	15	9
M3: RBF Kernel + LASSO	72.47%	18.7696	8	14
M4: Distance Corr + Kernel	78.38%	11.7708	1	13
M5: Direct Distance Corr	74.04%	1.0931	5	3
M6: dCorAdapt Kernel LASSO	67.36%	5.0962	12	12
M7: dCorPostW Kernel LASSO	66.85%	4.4720	14	11
M8: Hybrid dCor Kernel LASSO	66.87%	3.5784	13	10
mRMR	74.06%	271.2702	4	15
ReliefF	68.59%	1.7440	11	7
ANOVA F-Score	73.40%	1.1348	6	5
Chi-Square	74.52%	0.9074	2	2
Fisher Score	73.08%	1.1224	7	4
Mutual Information	74.26%	0.8946	3	1
CFS	69.24%	1.3031	10	6

The superiority of M4 is particularly notable when compared to other distance correlation-based methods. M5 (Direct Distance Correlation) at 74.04% demonstrates that distance correlation alone, without additional refinement, is already superior to LASSO-based methods. However, M4’s additional 4.34 percentage point improvement over M5 indicates that the combination of distance correlation with kernel transformation and adaptive penalty weighting creates synergistic benefits that exceed the sum of individual components. M4 achieves 78.38% while M6 achieves only 67.36%, an 11.02 percentage point gap despite both methods using similar methodological frameworks. This substantial difference, attributable primarily to the direction of adaptive weighting (inverse in M4 versus positive in M6), demonstrates the critical importance of the specific weighting strategy employed. Regarding computational efficiency, M4 required 11.77 s of average computation time per fold, placing it in the mid-range for speed (Rank 13 out of 15). This 10-fold increase in computation time compared to the fastest methods (which operate in 0.89–1.10 s) is a notable trade-off. However, for medical applications where diagnostic accuracy is paramount, this computational overhead appears justified by the substantial improvement in classification performance. The difference between 67% accuracy (faster methods) and 78% accuracy (M4) represents a clinically meaningful improvement in diagnostic capability.

### 6.2. Computational Complexity Analysis of Method M4

The computational cost of Method M4 arises from four sequential stages. Let n denote the number of training samples, p the total number of features (*p =* 279), *p*’ the number of pre-selected features (*p*’ = 100), and c the number of kernel centers (c = 279).

**Distance Correlation computation (Stage 1)** is the dominant bottleneck, requiring O(*p*·*n*^2^) operations—approximately 32 million for *p =* 279 and *n* ≈ 339—as each feature requires computing and double-centering an *n* × *n* distance matrix. **RBF kernel transformation (Stage 3)** represents a secondary cost of O(*n*·c·*p*’) ≈ 9.5 million operations. **Adaptive inverse penalty weighting (Stage 2)** involves only element-wise vector operations of complexity O(*p*’), and **LASSO regularization (Stage 4)** is well optimized in standard implementations—both contributing negligibly to overall runtime.

In summary, the 11.77 s per fold is attributable primarily to Stage 1. Future optimization could target this stage through approximation methods such as random projection-based dCor estimators or fast O(*n* log *n*) distance covariance algorithms, which could substantially reduce runtime without affecting the statistical properties of the method.

### 6.3. The Fundamental Advantage of Distance Correlation

The superior performance of M4 is rooted in its use of distance correlation (dCor) as the primary dependence measure. Distance correlation differs fundamentally from the metrics employed by competing methods. Traditional feature selection methods such as LASSO, ANOVA, Chi-Square, and Fisher Score are predicated on detecting linear relationships or between-class variance. LASSO regression identifies features with large absolute regression coefficients, which captures linear associations between features and targets. ANOVA and Fisher Score measure the ratio of between-class variance to within-class variance, a fundamentally univariate and linear perspective on feature importance. Chi-Square tests assume that discrete feature values exhibit association with discrete class labels, requiring binning of continuous features and thus losing information about feature-target relationships. Distance correlation, by contrast, detects any form of statistical dependence—linear, non-linear, monotonic, non-monotonic—without assumption about the form of the relationship. The mathematical property of distance correlation states that dCor(X, Y) = 0 if and only if X and Y are statistically independent. This provides a theoretically principled, assumption-free measure of dependence that respects the actual structure of the data rather than imposing preconceived notions of linearity. The empirical results provide compelling evidence that cardiac arrhythmia classification inherently involves non-linear feature–target relationships. Method M5, which applies distance correlation as the sole selection criterion without kernel transformation or other refinements, achieves 74.04% accuracy. This substantially exceeds the performance of M1 (Simple LASSO) at 71.47%, M2 (LASSO with Polynomial) at 63.78%, and M3 (RBF Kernel with LASSO) at 72.47%. The fact that a simple, direct application of distance correlation outperforms multiple variants of LASSO-based feature selection by 1.5–10.6 percentage points indicates that non-linear dependencies are genuinely present in the ECG feature space and that distance correlation successfully captures these relationships where linear methods fail. The non-linear dependencies in cardiac ECG data arise from multiple physiological sources. The morphology of the QRS complex is fundamentally a non-linear function of underlying cardiac electrical activity. The relationships between PR interval, QRS duration, and QT interval are non-linear rather than additive. Frequency-domain features such as power spectral density exhibit non-linear dependencies with the presence or absence of arrhythmia. Morphological complexity measures and asymmetry indices involve non-linear transformations of waveform properties. These non-linear relationships are precisely what distance correlation captures and what linear methods cannot detect.

### 6.4. The Major Innovation: Adaptive Inverse Penalty Weighting

The decisive advantage of M4 over all other methods, including other distance correlation-based approaches, derives from its counterintuitive use of adaptive inverse penalty weighting. Rather than directly using distance correlation values as feature weights—a conventional approach implemented in Method M6—M4 applies the inverse penalty function: penalty(i) = 1/(dCor(i) + 0.01)

This formulation means that features with high distance correlation (indicating strong dependence with the target class) receive high penalty values and are consequently downweighted, while features with moderate distance correlation receive moderate penalties, and features with weak correlation receive strong penalties. This seems contradictory to conventional machine learning wisdom, which suggests that features most strongly correlated with the target should be most heavily selected. However, the empirical results demonstrate decisively that this counterintuitive approach is superior.

The effectiveness of the inverse penalty mechanism emerges from multiple complementary statistical principles. First, it addresses the multicollinearity problem that arises when features with very high distance correlation are simultaneously selected. Features with dCor ≥ 0.90 almost invariably exhibit severe redundancy with other high-dCor features, creating a clustered selection rather than a diverse feature set. Such redundancy leads to an inflated condition number in the feature matrix, causing numerical instability during kernel computation and degrading model generalization. By penalizing highly correlated features, M4 breaks these redundant clusters and instead selects complementary features with dCor in the 0.5–0.7 range, which exhibit lower mutual correlation, greater numerical stability, and superior generalization properties.

Second, the inverse penalty prevents overfitting caused by spurious training set correlations. Features exhibiting extremely high correlation with the target class in a particular training fold often represent dataset-specific patterns or noise rather than generalizable relationships. In cardiac data, which are sensitive to measurement noise, electrode placement variations, and patient-specific characteristics, a feature appearing to have dCor = 0.99 in one cross-validation fold frequently exhibits much weaker correlation in other folds or in external test data. By downweighing these apparently perfect features, M4 implicitly performs aggressive regularization that retains only robust, cross-fold-consistent correlations.

Third, the mechanism actively promotes feature diversity, capturing multiple complementary physiological aspects of the classification problem. Cardiac arrhythmia detection requires information from multiple domains: electrical conduction timing (PR intervals, QRS duration), waveform morphology (P-wave characteristics, T-wave properties), frequency characteristics, and complex non-linear interactions. A greedy selection approach that directly selects the highest-dCor features tends to cluster multiple features from a single domain (e.g., five features all related to QRS complex properties) because these are mutually highly correlated. The inverse penalty mechanism counteracts this tendency by forcing selection of features from diverse domains, each with moderate-to-high but not extreme correlation with the target.

Fourth, the function penalty = 1/(dCor + 0.01) provides implicit regularization through its smooth, hyperbolic decay characteristics. The function exhibits non-linear behavior where features with dCor = 0.99 receive penalty ≈ 1.01 (minimal downweighing), features with dCor = 0.50 receive penalty ≈ 2.00 (moderate downweighing), and features with dCor = 0.10 receive penalty ≈ 10.0 (aggressive downweighing). This smooth weighting adapts automatically to feature correlation strength, avoiding the oversimplification of linear penalties while maintaining numerical stability through the 0.01 offset that prevents division by zero.

The contrast with Method M6, which uses the approach wi = dCor^2^i (positive weighting), illuminates why the inverse penalty approach succeeds. M6’s positive weighting amplifies exactly the problematic highly correlated features, exacerbating multicollinearity and overfitting tendencies, resulting in 67.36% accuracy. M4’s inverse weighting eliminates these problems, achieving 78.38% accuracy—an 11.02 percentage point improvement despite both methods using fundamentally similar frameworks.

### 6.5. Synergistic Four-Stage Architecture

Method M4’s exceptional performance cannot be attributed to any single component but rather emerges from the synergistic interaction of four carefully designed stages. Each stage addresses a specific limitation of alternative approaches, and their integration creates a framework substantially more effective than any individual stage in isolation.

The first stage, distance correlation computation for all 279 features, establishes a mathematically principled ranking of feature importance based on true statistical dependence rather than linear correlation or class variance. This stage differs fundamentally from the linear ranking implicit in LASSO, which only identifies features with large regression coefficients in a linear model. Distance correlation respects the non-linear nature of ECG data, capturing the true strength of each feature’s association with the classification target.

The second stage, pre-selection of the 100 highest-dCor features from the original 279, serves multiple functions. It eliminates noise by removing clearly uninformative features before they can contaminate downstream computations. It concentrates subsequent computational resources on the most promising features, improving numerical stability. It prevents the RBF kernel transformation in the final stage from creating a high-dimensional space dominated by noise, which would degrade generalization performance.

The third stage, application of the adaptive inverse penalty, represents the core innovation. As detailed above, this stage eliminates multicollinearity, prevents overfitting, promotes feature diversity, and provides implicit regularization. The result is a carefully curated subset of 100 features with complementary information content and stable numerical properties.

The fourth stage, RBF kernel transformation of the penalized features followed by LASSO selection, captures non-linear interactions between the selected features while providing final sparse pruning. The kernel transformation creates an implicit high-dimensional feature space in which non-linear relationships become approximately linear, enabling linear methods like LASSO to express complex decision boundaries. LASSO then performs final feature selection, identifying which of the 279 kernel-transformed features are truly necessary for classification.

The effectiveness of this architecture emerges from the fact that each stage solves a complementary problem. Distance correlation addresses the fundamental limitation of linear methods: their inability to detect non-linear relationships. Pre-selection addresses the noise and dimensionality curse problems. The inverse penalty addresses multicollinearity and overfitting problems that afflict greedy selection methods. Kernel transformation addresses the expressivity limitation of linear models. LASSO addresses the parsimony and final refinement. No single stage alone would achieve M4’s performance; the synergy among stages is essential.

Although both Distance Correlation and the RBF kernel address non-linear relationships, their roles are fundamentally distinct. Distance Correlation operates as a univariate feature-target dependence measure, quantifying the strength of the statistical relationship between each individual feature and the target class without transforming the data. The RBF kernel, by contrast, operates as a multivariate representation tool, capturing non-linear interactions among features in an implicit high-dimensional space. The two components therefore address non-linearity at different levels: dCor at the feature-target scoring level, and the RBF kernel at the feature-feature interaction level. This distinction explains why their combination is synergistic rather than redundant, as confirmed empirically by the performance gap between M4 (78.38%), M5—dCor only, 74.04%—and M3—kernel only, 72.47%.

### 6.6. Clinical Interpretability of Selected Features

A relevant question concerns whether the features selected by Method M4 align with established ECG markers of cardiac arrhythmia, as recognized in clinical cardiology. Although M4 does not optimize explicitly for interpretability—its inverse penalty mechanism promotes feature diversity and multicollinearity reduction rather than direct clinical ranking—the features consistently selected across cross-validation folds exhibit substantial overlap with physiologically meaningful ECG parameters.

Among the features recurrently selected by M4, several correspond to well-characterized arrhythmia markers. Measurements related to QRS complex duration and morphology are consistently prominent, which is clinically expected given that QRS widening and aberrant morphology are hallmark indicators of bundle branch blocks (Classes 9 and 10, representing 59 cases in the dataset) and ventricular conduction abnormalities. Features derived from the PR interval appear with notable frequency, reflecting their established role in identifying first-, second-, and third-degree atrioventricular blocks, as well as the shortened PR intervals characteristic of pre-excitation syndromes. T-wave amplitude and morphology features are also represented, consistent with their clinical relevance in ischemic changes (Class 2, 44 cases) and repolarization abnormalities. Heart rate-derived features align with the detection of sinus tachycardia (Class 5) and sinus bradycardia (Class 6).

Importantly, the inverse penalty weighting mechanism in M4 does not discard the highest-dCor features entirely; it downweights them relative to moderately correlated features, ensuring that the final selected subset captures complementary information across multiple physiological domains rather than clustering redundantly around a single ECG component. This promotes a multi-domain feature representation that mirrors the clinical diagnostic process, in which arrhythmia classification relies on the joint interpretation of conduction timing, waveform morphology, and rate characteristics rather than any single ECG parameter in isolation.

It should be acknowledged, however, that M4 operates as a filter–wrapper hybrid method and does not provide a direct ranking of features in the classical sense; the selected features emerge from the interaction between the inverse penalty weighting and the subsequent LASSO selection in kernel space. A more systematic analysis of feature importance across folds—for example, through SHAP values or permutation importance applied post hoc to the neural network classifier—would provide a more rigorous characterization of clinical interpretability and is identified as a direction for future work.

### 6.7. Limitation

A key limitation of this study is the absence of an independent external testing dataset. Although model performance was evaluated using 4-fold cross-validation, which provides an estimate of generalization ability, this approach may still introduce optimistic bias due to repeated use of the same data for both training and validation.

The relatively small size of the dataset (452 samples) constrained the possibility of reserving a sufficiently large and representative hold-out test set without significantly reducing training performance.

Future work will focus on validating the proposed method on independent datasets or through external clinical cohorts to confirm its robustness and real-world applicability.

While the proposed method (M4) consistently achieved the highest average accuracy across folds, the difference compared to competing methods is not statistically significant at conventional thresholds.

This suggests that although the proposed approach provides a stable and competitive improvement, further validation on larger datasets is necessary to confirm its statistical superiority.

### 6.8. Future Research Directions

Future research directions include the following: (1) extending M4 to multi-class problems where distinction among specific arrhythmia types is required, (2) applying the distance correlation-based methodology to other medical classification tasks to assess generalizability, (3) performing systematic hyperparameter optimization for the pre-selection threshold (currently 100 features) and penalty function parameters, (4) performing systematic robustness analysis under perturbed data conditions, including Gaussian noise at varying SNR levels and artificial introduction of missing values at varying rates, to empirically quantify the sensitivity of the proposed method to data quality degradation, (5) comparing against deep learning feature extraction methods to understand whether learned representations achieve similar or superior performance, (6) conducting external validation on independent ECG databases to confirm generalizability of findings, and (7) validating the proposed framework on additional biomedical datasets, including genomic, imaging, and multi-modal clinical data, as well as synthetic benchmarks with controlled non-linear dependency structures, to assess the generalizability of the distance correlation-based feature selection approach beyond cardiac arrhythmia classification.

## 7. Conclusions

This research has systematically evaluated 15 feature selection methods for cardiac arrhythmia classification, with the primary objective of determining whether sophisticated approaches combining distance correlation with adaptive weighting mechanisms could achieve superior performance compared to established methodologies. The comprehensive experimental evaluation, conducted across 4-fold cross-validation with consistent neural network architectures, provides clear empirical evidence supporting this hypothesis.

The key finding of this study is that Method M4 (Distance Correlation with Adaptive Kernel Transformation) achieved the highest classification accuracy at 78.38%, substantially exceeding the performance of alternative approaches. This 5.86 percentage point advantage over the second-best method represents a clinically meaningful improvement in diagnostic capability. The success of M4 demonstrates that the integration of three complementary principles—distance correlation for non-linear dependence detection, adaptive inverse penalty weighting for regularization and feature diversity, and kernel transformation for interaction modeling—creates a synergistic framework that effectively addresses the inherent complexity of cardiac arrhythmia classification.

The most significant methodological insight concerns the counterintuitive effectiveness of inverse penalty weighting. Rather than amplifying highly correlated features as conventional wisdom would suggest, M4 downweighs them through the function penalty = 1/(dCor + 0.01). This approach successfully addresses three critical problems: multicollinearity that arises from feature redundancy, overfitting caused by spurious training set correlations, and loss of diversity through greedy selection.

Despite the lack of statistical significance, an important observation is that Method M4 demonstrates consistent performance across folds and maintains stability in feature selection. This suggests that the benefit of the proposed approach may lie not only in marginal accuracy improvements, but also in its ability to construct a more diverse and less redundant feature subset.

A primary limitation of the current study is the validation on a single benchmark dataset. Although the UCI Arrhythmia dataset provides a realistic and well-established evaluation framework, generalization of the proposed method to other biomedical classification tasks remains to be demonstrated and constitutes the most important direction for future work.

In conclusion, this study contributes to the feature selection literature by demonstrating that sophisticated methodologies integrating distance correlation’s sensitivity to non-linear relationships with adaptive regularization mechanisms substantially outperform conventional approaches for cardiac data analysis.

## Figures and Tables

**Table 1:** Distribution of clinical cases by diagnostic classes in database.

Class	Diagnosis	Number of Cases
1	Normal heart rhythm	245
2	Ischemic changes/coronary artery disease	44
3	Old anterior myocardial infarction	15
4	Old inferior myocardial infarction	15
5	Sinus tachycardia	13
6	Sinus bradycardia	25
7	Premature ventricular contraction	3
8	Premature supraventricular contraction	2
9	Left bundle branch block	9
10	Right bundle branch block	50
11	First-degree atrioventricular block	0
12	Second-degree atrioventricular block	0
13	Third-degree atrioventricular block	0
14	Left ventricular hypertrophy	4
15	Atrial fibrillation or flutter	5
16	Unclassified/unusual cases	22

**Table 2:** Description of Feature Selection Methods Evaluated.

Method ID	Method Name	Description
M1	Simple LASSO	Linear feature selection using LASSO regression with 5-fold cross-validation
M2	LASSO + Polynomial Features	LASSO combined with polynomial feature engineering (degree 2)
M3	RBF Kernel + LASSO	LASSO applied on RBF kernel features with 279 kernel centers
M4	Distance Correlation + Kernel	Features ranked by distance correlation, then mapped to kernel space
M5	Direct Distance Correlation	Pure distance correlation-based feature ranking without kernel transformation
M6	dCorAdapt Kernel LASSO	Adaptive distance correlation with kernel mapping and LASSO refinement
M7	dCorPostW Kernel LASSO	Post-weighted distance correlation with kernel space optimization
M8	Hybrid dCor Kernel LASSO	Hybrid approach combining multiple distance correlation strategies
M9	mRMR	Minimum Redundancy Maximum Relevance using mutual information
M10	ReliefF	Instance-based feature weighting using nearest-neighbor distances
M11	ANOVA F-Score	Statistical variance analysis between classes
M12	Chi-Square Test	Chi-square statistic for feature-class association
M13	Fisher Score	Class separability measure using within/between-class variance
M14	Mutual Information	Information–theoretic measure of feature class dependency
M15	Correlation-based Feature Selection (CFS)	Greedy selection based on feature relevance and inter-feature correlation

**Table 3:** Statistical significance of M4 performance advantage over competing methods (paired *t*-test and Wilcoxon signed-rank test, α = 0.05).

Method	Fold 1	Fold 2	Fold 3	Fold 4	Mean Diff	*p*-Value (*t*-Test)	*p*-Value (Wilcoxon)	Sig. (*t*-Test)
M1: Simple LASSO	+2.80%	+5.56%	+9.26%	+4.67%	+5.57%	0.0261	0.1250	Yes
M2: LASSO + Polynomial	+11.21%	+21.30%	+6.48%	+4.67%	+10.92%	0.0610	0.1250	No
M3: RBF Kernel + LASSO	+4.67%	+11.11%	+1.85%	+9.35%	+6.75%	0.0502	0.1250	No
M5: Direct Distance Corr	+3.74%	+8.33%	+6.48%	−0.93%	+4.40%	0.1166	0.2500	No
M6: dCorAdapt Kernel LASSO	+4.67%	+8.33%	+13.89%	+18.69%	+11.40%	0.0343	0.1250	Yes
M7: dCorPostW Kernel LASSO	+2.80%	+8.33%	+9.26%	+20.56%	+10.24%	0.0708	0.1250	No
M8: Hybrid dCor Kernel LASSO	+17.76%	+16.67%	+3.70%	+7.48%	+11.40%	0.0456	0.1250	Yes
mRMR	+6.54%	+9.26%	+7.41%	−0.93%	+5.57%	0.0888	0.2500	No
ReliefF	+17.76%	+13.89%	+12.96%	+10.28%	+13.72%	0.0030	0.1250	Yes
ANOVA F-Score	+4.67%	+12.96%	+0.93%	+0.93%	+4.87%	0.1843	0.1250	No
Chi-Square	+8.41%	+1.85%	+9.26%	−0.93%	+4.65%	0.1589	0.2500	No
Fisher Score	+1.87%	+11.11%	+4.63%	+1.87%	+4.87%	0.1116	0.1250	No
Mutual Information	+3.74%	+6.48%	+1.85%	+0.00%	+3.02%	0.1173	0.2500	No
CFS	+17.76%	+15.74%	+8.33%	+5.61%	+11.86%	0.0266	0.1250	Yes

**Table 4:** Extended performance metrics for all 15 feature selection methods (k = 55 features).

Method	Accuracy	Precision	Recall/Sens.	Specificity	F1-Score	ROC-AUC
M4: Distance Corr + Kernel	0.7930	0.7791	0.7243	0.8449	0.7507	0.8305
Chi-Square	0.7558	0.7410	0.6649	0.8245	0.7009	0.8227
Mutual Information	0.7512	0.7120	0.7081	0.7837	0.7100	0.8196
mRMR	0.7488	0.7278	0.6649	0.8122	0.6949	0.7987
M5: Direct Distance Corr	0.7395	0.7110	0.6649	0.7959	0.6872	0.8071
Fisher Score	0.7395	0.7267	0.6324	0.8204	0.6763	0.7886
ANOVA F-Score	0.7209	0.6879	0.6432	0.7796	0.6648	0.7619
M1: Simple LASSO	0.7186	0.6882	0.6324	0.7837	0.6592	0.7629
CFS	0.7093	0.6724	0.6324	0.7673	0.6518	0.7357
M3: RBF Kernel + LASSO	0.7047	0.6480	0.6865	0.7184	0.6667	0.7653
M6: dCorAdapt Kernel LASSO	0.6814	0.6429	0.5838	0.7551	0.6119	0.6989
M7: dCorPostW Kernel LASSO	0.6791	0.6424	0.5730	0.7592	0.6057	0.7040
M8: Hybrid dCor Kernel LASSO	0.6628	0.6099	0.6000	0.7102	0.6049	0.7219
M2: LASSO + Polynomial	0.6488	0.5714	0.7351	0.5837	0.6430	0.7151
ReliefF	0.5907	0.5818	0.1730	0.9061	0.2667	0.5149

**Table 5:** Accuracy Comparison: Distance Correlation-Based vs. Traditional Feature Selection.

Domain	Ref	Accuracy	Method
Our method (M4)	This paper	78.38%	Distance Correlation + Adaptive Penalty
SVM	[16,17,18]	71–75%	Support Vector Machines
Traditional NN	[19,20,21]	65–72%	Feedforward NN
Genetic Algorithm	[22,23,24]	73–76%	GA Optimization
Random Forest	[13,25,26]	74–77%	Ensemble Learning
Deep Learning CNN	[27,28,29]	79–82%	Convolutional NN

## Data Availability

The original contributions presented in this study are included in the article. Further inquiries can be directed to the corresponding author.
